# Machine Learning-Based Comparative Analysis of Subject-Independent EEG Data Classification Across Multiple Meditation and Non-Meditation Sessions

**DOI:** 10.3390/s25226876

**Published:** 2025-11-11

**Authors:** Nalinda D. Liyanagedera, Corinne A. Bareham, Heather Kempton, Hans W. Guesgen

**Affiliations:** 1 School of Mathematical and Computational Sciences, Massey University, Palmerston North 4410, New Zealand; h.w.guesgen@massey.ac.nz; 2 Department of Computing & Information Systems, Faculty of Applied Sciences, Wayamba University of Sri Lanka, Kuliyapitiya 60200, Sri Lanka; 3 School of Psychology, Massey University, Palmerston North 4410, New Zealand; c.bareham-waldock@massey.ac.nz; 4 School of Psychology, Massey University, Auckland 0632, New Zealand; h.kempton@massey.ac.nz

**Keywords:** EEG (Electroencephalography), BCI (brain computer interface), machine learning, subject independence, classification, meditation, multiple session, CSP (Common Spatial Pattern), STFT (Short-Time Fourier Transform), neural network

## Abstract

In this study, subject-independent (inter-subject), multiple-session electroencephalography (EEG) data classification was tested for loving-kindness meditation (LKM) and non-meditation. This is a novel study that extends our previous work on intra-subject, multiple-session classification. Here, two meditation techniques, LKM-Self and LKM-Other, were independently compared with non-meditation. For each mental task, five sessions of data collected from each of the twelve participants were placed in a common data pool, from which randomly selected session data were used for training and testing the machine learning algorithms. Three previously tested BCI pipelines were used. In each case, feature extraction was performed using common spatial patterns (CSPs), short-time Fourier transform (STFT), or a fusion of CSP and STFT, followed by classification using a neural network structure. This study was further divided into three cases, where two, three, or four session pairs were used to train the algorithms, and the remaining session pair was used for testing. For each individual instance, the test was repeated thirty times to generalize the results. Thus, a total of 9900 independent tests were conducted for the entire dataset. The mean classification accuracies obtained in this study were lower than those reported in our previous intra-subject classification study. For example, in LKM-Self/non-meditation classification using three session pairs with the CSP + STFT pipeline, the mean accuracy for all tests was 62.3%, with the bottom 50% at 46.0% and the top 50% at 78.3%, demonstrating variability across session selections. The corresponding intra-subject classification result for the same instance was 72.1%. For all other instances, a similar pattern was observed. Furthermore, when considering all mean accuracies obtained, in 83.3% of the instances, CSP + STFT produced better classification accuracies than CSP or STFT alone. At the same time, in 75.0% of the instances, increasing the number of training session pairs led to improved classification accuracies. This study demonstrates that the subject-independent, multiple-session EEG classification of meditation and non-meditation is feasible for specific session combinations. Further research is needed to confirm these findings across larger and more diverse participant groups. These findings provide a foundation for developing subject-independent algorithms that can guide long-term meditation practice.

## 1. Introduction

Electroencephalography (EEG) has emerged as a highly effective non-invasive method for measuring human cognitive activities and mental states, with applications ranging from neurorehabilitation to mindfulness training [1,2]. In recent years, advances in sensor technology and machine learning have supported the development of systems that interpret brain activity patterns, particularly those that aim to achieve subject-independent analysis [3,4,5]. Among these applications, meditation assessment has received significant attention due to its relevance to mental health [6], stress reduction [7], and cognitive enhancement [1,8]. However, a major challenge in developing generalized EEG-based systems for such purposes lies in the variability of brain signals across individuals and sessions [9]. This study focuses on the subject-independent classification of EEG signals across multiple meditation and non-meditation sessions using machine learning techniques, with the goal of improving the robustness and reliability of EEG-based mental state monitoring systems [10,11].

Meditation has been proven to cause both temporary and permanent changes in a person [12,13], where “Trait” [14,15] refers to the lasting changes that develop after long-term meditation practice. “State” [16,17], on the other hand, refers to the temporary changes that occur while a person is meditating compared to when they are not. Since this study aims to classify meditation and non-meditation states, we focus on “State” characteristics. Many people who practise meditation aim to achieve progressively calmer mental states, and currently, psychological assessments such as mindfulness scales, questionnaires, and interviews have demonstrated such progressive differences among individuals who practise meditation [18,19].

At the same time, neuroimaging techniques such as EEG [20,21,22], fMRI (functional magnetic resonance imaging) [23], fNIRS (functional near-infrared spectroscopy) [24], and PET (positron emission tomography) [25], along with physiological measurements such as heart rate variability [26] and cortisol levels [27], have been used to understand various characteristics of meditation/non-meditation. A significant number of studies have been conducted using EEG to examine different aspects of meditation/non-meditation [28,29,30], and some of these studies highlight the importance of developing software systems that can support individuals in practicing meditation, particularly in guiding them toward progress [31,32,33]. At the most basic level, the calmness achieved through meditation can be identified by comparing brain patterns during meditation and non-meditation, with non-meditation serving as a baseline [29,34]. However, since meditation skills improve over time with repeated practice, understanding states such as calmness requires computational analysis and recognition of patterns in multiple-session meditation/non-meditation EEG data [35,36].

We recognized the importance of studying patterns in multiple-session meditation/non-meditation EEG data [37,38,39]. Our earlier work [37] showed that while meditation sessions share similar EEG characteristics, they are clearly distinguishable from non-meditation sessions. As a next step, in our following study [38], we successfully classified intra-subject, multiple-session meditation/non-meditation EEG data. When developing a system that can monitor and distinguish brain pattern differences between meditation and non-meditation EEG data, it is more practical and user-friendly to design it as a subject-independent system, since such systems do not require personal calibration or prior data collection from each individual user, making them easier to use and more accessible in real-world applications. Therefore, as the next step in our study, in this paper, we plan to test subject-independent, multiple-session meditation/non-meditation EEG data classification and compare this performance with the outcomes of our previous intra-subject classification study [38].

EEG is a method used to measure the electrical activity of the brain [40,41] by placing electrodes on the scalp at predefined locations [42]. Studies have shown that EEG signals collected in this manner, which consist of vibration-like patterns, can correlate with different mental tasks [43]. One common method for analyzing EEG data is to first transform it into the frequency domain [44], where, based on specific characteristics, the signal is divided into five frequency bands: Delta (0.5–4 Hz), Theta (4–8 Hz), Alpha (8–13 Hz), Beta (13–30 Hz), and Gamma (>30 Hz) [45,46,47]. Based on past research, the Theta [48,49,50,51] and Alpha [52,53,54] bands are most commonly associated with meditation/non-meditation studies, and they were utilized in our study as well. EEG mainly consists of non-linear and complex data; therefore, extracting meaningful patterns from it is a lengthy and challenging process, commonly referred to as the brain–computer interface (BCI) pipeline [55].

A BCI pipeline consists of multiple steps that can generally be broken down into data collection, preprocessing, feature extraction, classification, and application [56,57]. Preprocessing involves cleaning and artifact removal of the collected EEG data and modifying it into a different structure or a simpler format, such as through dimensionality reduction [58,59]. Since EEG data are complex and have a low signal-to-noise ratio, this cleaning and preprocessing step plays a significant role in the entire study. After the data is cleaned and converted into the desired format, the next step is feature extraction [56,60]. The aim here is to extract important information hidden in the EEG data that can be used for valuable future tasks. Even after thorough cleaning, a certain amount of noise still remains in the EEG data. Because of this, advanced signal processing methods such as short-time Fourier transform (STFT) [61,62], common spatial patterns (CSPs) [63,64,65], and event-related potentials (ERPs) [66] are used in feature extraction.

After feature extraction, the next step in the BCI pipeline is classification and application [67,68,69]. When we examine past work, notable classification algorithms used with meditation EEG include Support Vector Machines (SVMs) [70], Linear Discriminant Analysis (LDA) [71], Artificial Neural Networks (ANNs) [72], Decision Trees, Random Forests [4], and k-Nearest Neighbors (k-NN) [9]. Among these, SVMs and ANNs were the most commonly used methods, with ANNs being significantly utilized by researchers in the recent past. The use of ANNs has increased not only with EEG but also in many research scenarios involving large, nonlinear datasets with high computational complexity that require processing to extract meaningful patterns [73]. This is because ANNs can function as deep learning algorithms that learn from data, adapt to new information, and model complex nonlinear relationships [74]. An ANN structure behaves similarly to neuron interactions in the brain, and a basic ANN consists of an input layer, an output layer, and a number of hidden layers, where each layer contains one or more nodes depending on the problem it is designed to solve. An ANN is first trained on a dataset before being used on a new dataset, and training occurs by sending signals forward and backward through the network while optimizing the internal weights to learn from the training data [75]. After a significant amount of analysis, in our previous study on intra-subject multiple-session classification, we used ANNs to ensure consistent conditions during classification while comparing the performance of different feature extraction methods [38]. In this study, we intend to use the proven BCI pipelines from our previous study, allowing us to compare the performance of intra-subject and inter-subject multiple-session meditation/non-meditation classification on common ground.

This study aims at the novel task of evaluating how effectively subject-independent EEG data classification can be achieved for multiple-session meditation and non-meditation EEG data. This work extends our previously published study [38], where we first demonstrated that multiple-session meditation and non-meditation EEG data exhibit common characteristics within each group. Following that, we successfully performed intra-subject classification of multiple-session meditation and non-meditation EEG data. As a continuation of these studies, this work focuses on subject-independent classification of multiple-session meditation and non-meditation EEG data.

By focusing on both multiple-session meditation and subject-independent classification, this study adds significant value to scientific research. Past studies show a growing demand and interest in achieving subject-independent EEG data classification, particularly in areas such as motor imagery [56,76]. Such an advancement would enable the development of applications that can be trained on existing data and used by new individuals without further modifications. Unfortunately, no prior research exists on subject-independent, multiple-session meditation EEG classification. Therefore, this study represents a key milestone in initiating and encouraging the development of algorithms that can support apps guiding individuals in their meditation progress. The value of such an app is particularly significant when it is subject-independent and supports multiple-session meditation practice, as true progress in meditation can only be achieved through consistent long-term practice.

## 2. Materials and Methods

This study is an extension of our previous work [38], where we successfully demonstrated intra-subject, multi-session EEG data classification for meditation and non-meditation states. In this work, we test how subject-independent, multi-session EEG data classification for meditation and non-meditation states performs compared to our previous study. Since we aim to compare the results of inter-subject classification with our earlier intra-subject classification, this study follows a procedure similar to our previous work, where the only difference is that, instead of using EEG sessions from the same person for training and testing on the selected machine learning algorithms, EEG sessions from different participants were used. Since the data preprocessing and construction of the BCI pipelines were carried out similarly to our previous study, only the most important steps are summarized here, and readers can refer to our earlier paper [38] for the full description. At the same time, the parts of the procedure that are unique to this study are thoroughly explained in this section.

This study uses an EEG dataset available online, with the dataset’s DOI as follows: https://doi.org/10.18112/openneuro.ds003816.v1.0.1. A description of the dataset can be found in one of our previous research articles [37]. The dataset is labelled as experienced meditators [77,78,79], but since there is no measurement of experience levels, such as years of practice, we assume that there may be varying levels of experience among the expert participants. In this study, five sessions of EEG data collected per mental task for each of the 12 participants were used, which is a subset of the above online dataset. The study uses both meditation and non-meditation EEG data. Two meditation types were used in this study: loving-kindness meditation [80,81] on oneself (LKM-Self) and on others (LKM-Others). Therefore, for each participant, 15 sessions of EEG data were used, with 5 sessions per meditation type and 5 sessions of non-meditation data. Two independent classification tests were conducted: one for LKM-Self vs. non-meditation and the other for LKM-Others vs. non-meditation. Altogether, 180 (15 × 12) sessions of EEG data were used in this study, and these 180 sessions were the highest-quality sessions selected from the original dataset after initial cleaning and screening.

The selected 180 sessions of EEG data were further cleaned and preprocessed using EEGLAB [59,82] in MATLAB R2021b. A “Basic FIR filter” was applied to extract the data between 2 Hz and 45 Hz, and bad channels were identified and corrected [83,84]. Independent component analysis (ICA) [71,85,86] was then performed on each EEG data session, and cleaning was carried out by selecting the appropriate components through visual inspection [59,87]. Since the original EEG data had 127 channels, principal component analysis (PCA) [71,85] was used along with ICA to achieve dimensionality reduction [88,89], allowing ICA to run smoothly. The cleaned EEG data sessions were saved in “.eeg” file format for use in the BCI pipelines developed using Python 3.9 [90,91].

In our previous study [38], intra-subject classification was performed using 5 session pairs of EEG data for the two compared meditation/non-meditation mental tasks for a selected participant. Since we plan to compare the results of the two studies, in this study, we also used 5 session pairs of EEG data for the mental tasks. After selecting these 10 sessions, we used three sizes of training data to match our previous study in order to compare the results. Therefore, in this study, we used 2 session pairs, 3 session pairs, or 4 session pairs of EEG data for training the machine learning algorithms in independent analyses.

The cleaned data consists of 180 sessions from 12 participants. Since we are working on inter-subject classification, we first modelled this dataset into three groups corresponding to the three mental tasks. Specifically, 60 sessions of data were assigned to LKM-Self, LKM-Others, and non-meditation, with each group containing 5 sessions of EEG data from a single participant. Two independent studies were conducted to classify LKM-Self vs. non-meditation and LKM-Others vs. non-meditation. Since the procedures were the same for both cases, the description is provided only for the LKM-Self vs. non-meditation classification.

For all independent tests conducted, the first task was to randomly select 5 participants per mental task, ensuring that each participant contributed at most one session for that task. Since each participant had 5 sessions of data per mental task, 1 session was then randomly selected from the available 5 for each chosen participant. In this way, each mental task contained data from 5 different participants. Among the two mental tasks, a single participant’s data might appear in one or both tasks depending on the random selection. The selected 5 meditation and 5 non-meditation sessions were then paired randomly to create 5 meditation/non-meditation session pairs. This selection process was repeated each time a pair of meditation/non-meditation mental tasks was used to study their classification strength.

For the LKM-Self vs. non-meditation pair, three independent studies were conducted, each using a different number of training session pairs. These studies were evaluated using three independent BCI pipelines. In this study, we used the same three BCI pipelines that produced the best performance in our previous work [38] on intra-subject multiple-session meditation/non-meditation EEG classification. Since, as part of our study, we intend to compare the performance of intra-subject and inter-subject classifications, we used the same BCI pipelines. Here, each of these EEG session data was broken down into epochs of 2 s in size with a 1 s overlap [92]. The main difference among these three BCI pipelines is the method used for feature extraction. The three BCI pipelines use either common spatial patterns (CSPs) [63,64,65], short-time Fourier transform (STFT) [61,62], or a fusion of CSP and STFT in each pipeline for feature extraction.

To give some background on the feature extraction methods used in the three BCI pipelines, common spatial patterns (CSPs) identify spatial filters that maximize variance differences between two mental tasks, capturing discriminative patterns across EEG channels. Short-time Fourier transform (STFT) analyzes the spectral content of EEG signals over short time windows, allowing the extraction of frequency-based features such as theta and alpha bands, which are relevant to meditation. The third pipeline combines CSP and STFT, taking advantage of both spatial and spectral information and providing a richer feature representation for classification. These methods are critical for the current study, as they enable the BCI pipelines to extract complementary information from multi-session EEG data and differentiate meditation from non-meditation states effectively.

In this study, EEG data were analyzed across several frequency ranges to identify the most effective band for meditation and non-meditation classification. Consistent with our previous findings [38], the theta and alpha frequency range (4–13 Hz) again produced the best classification performance. In all three BCI pipelines, artificial neural networks [74,75] were used as the classification algorithms, providing a common ground to compare the performance of the three feature extraction algorithms. We tested several network configurations and activation functions, and similarly to our previous study, a compact multi-layer perceptron (MLP) with two hidden layers of 20 nodes and a logistic activation function provided the best performance. Although EEG data are high-dimensional, dimensionality-reduction techniques reduced the feature set to a maximum of 14 inputs for each BCI pipeline. For this compact input, the two 20-node hidden-layer structure offered the highest accuracy with low computational cost, while the increasing network size did not improve performance. Since we used three different training sizes on three different BCI pipelines, we conducted nine different studies. Also, since this was carried out for the classification of LKM-Self vs. non-meditation and LKM-Others vs. non-meditation independently, this adds up to eighteen different studies that we conducted.

All the tests conducted started with a random selection of 5 pairs of EEG session data for the selected meditation/non-meditation mental tasks. Then, three different studies were conducted based on the number of session pairs used for the training of the machine learning algorithms. In the following sections, each of these three studies is explained one after the other.

The first study was conducted using 2 EEG data session pairs for training and 1 EEG data session pair for testing the BCI pipelines. This study starts by randomly selecting 5 EEG data session pairs for the LKM-Self and non-meditation mental tasks. In this study, 3 out of 5 session pairs were selected at a time, with 2 pairs used for training and the remaining session pair used for testing. Here, interchanging the testing session pair gave 3 tests for the 3 selected session pairs. Using the 5 selected pairs, multiple tests were conducted on various selection combinations of 3 session pairs out of 5. With different selection combinations, a total of 30 tests were conducted for each randomly selected 5-session pair of LKM-Self/non-meditation. This random selection of 5 session pairs of EEG data for LKM-Self and non-meditation was repeated for 30 independent experiments, resulting in 900 tests. Since 900 tests were conducted on one type of BCI pipeline and we used 3 independent BCI pipelines in our study, the total number of tests conducted was 2700. Therefore, for these 3 BCI pipelines, a total of 2700 (900 × 3) tests were conducted on the classification of LKM-Self vs. non-meditation using 3 session pairs of EEG data in the study.

The second study was conducted using 3 EEG data session pairs for training and 1 EEG data session pair for testing the BCI pipelines. This study begins by randomly selecting 5 EEG data session pairs for the LKM-Self and non-meditation mental tasks. In this study, 4 out of the 5 session pairs were selected at a time, with 3 pairs used for training and the remaining session pair used for testing. Here, interchanging the testing session pair gave 4 tests for the selected 4 session pairs. Using the selected 5 pairs, multiple tests were conducted on various selection combinations of 4 session pairs out of 5. With different selection combinations, a total of 20 tests were conducted for each randomly selected 5-session pair of LKM-Self/non-meditation. This random selection of 5 session pairs of EEG data for LKM-Self and non-meditation was repeated for 30 independent experiments, resulting in 600 tests. Since 600 tests were conducted on one type of BCI pipeline, and we used 3 independent BCI pipelines in our study, the total number of tests conducted was 1800. For these 3 BCI pipelines, a total of 1800 (600 × 3) tests were conducted on the classification of LKM-Self vs. non-meditation using 4 session pairs of EEG data in the study.

The third study was conducted using 4 EEG data session pairs for training and 1 EEG data session pair for testing the BCI pipelines. This study begins by randomly selecting 5 EEG data session pairs for the LKM-Self and non-meditation mental tasks. In this study, out of the 5 session pairs selected at a time, 4 pairs were used for training, and the remaining session pair was used for testing. Here, interchanging the testing session pair gave 5 tests for the randomly selected 5 session pairs of LKM-Self/non-meditation. This random selection of 5 session pairs of EEG data for LKM-Self and non-meditation was repeated for 30 independent experiments, resulting in 150 tests. Since 150 tests were conducted on one type of BCI pipeline, and we used 3 independent BCI pipelines in our study, the total number of tests conducted was 450. For these 3 BCI pipelines, hence, 450 (150 × 3) tests were conducted on the classification of LKM-Self vs. non-meditation using 5 session pairs of EEG data in the study.

**Figure 1:**
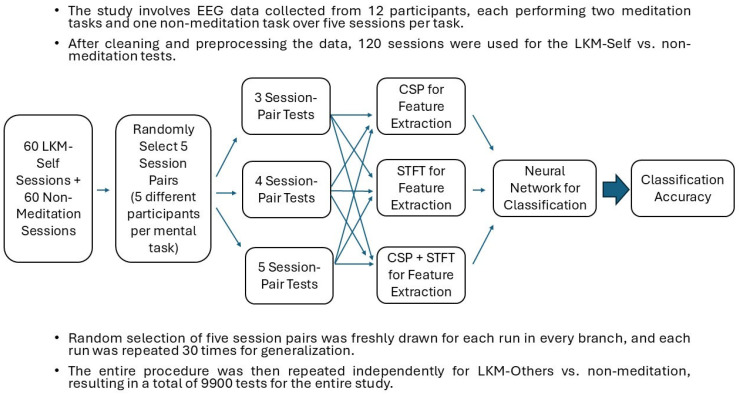
Workflow of inter-subject EEG classification across multiple meditation and non-meditation sessions with different training–testing setups.

When considering all three studies with different numbers of session pairs tested on the 3 BCI pipelines, a total of 4950 (2700 + 1800 + 450) tests were conducted to study the classification of LKM-Self vs. non-meditation. A similar approach was used to study the classification of LKM-Others vs. non-meditation; thus, a total of 9900 (4950 × 2) independent tests were conducted in our study for testing inter-subject multiple session meditation and non-meditation EEG data classification. The full description of the implementation of the 3 BCI pipelines is provided in our previous research article [38], and a summary of the procedure is shown in Figure 1. Our work clearly describes the steps for using CSP, STFT and the fusion of CSP and STFT for feature extraction. It also describes how deep learning was achieved using artificial neural networks with 2 hidden layers to match each of the feature sets and obtain optimal outcomes.

## 3. Results

In this study, we evaluated the effectiveness of subject-independent multiple-session EEG data classification for distinguishing between meditation and non-meditation states. We plan to compare these findings with our previous research on intra-subject multiple-session EEG data classification. The results are mainly divided into two parts, as we independently compared non-meditation with two meditation techniques: LKM-Self and LKM-Others. Each of these comparisons was tested under three conditions, based on the number of EEG session pairs used, specifically three, four, and five session pairs for both LKM-Self/non-meditation and LKM-Others/non-meditation classification tasks.

As a result, six tables were generated to present the findings from these six studies. For each study, three independent classification tests were conducted using three different BCI pipelines, which employed CSP, STFT, or a fusion of CSP and STFT for feature extraction. Accordingly, each of the six tables includes the classification accuracies achieved by all three pipelines. Each study was repeated independently 30 times to generalize the findings and improve result reliability. Leave-one-session-out cross-validation was applied, and the average and standard deviation were computed to report the classification accuracy and associated uncertainty for each test.

Table 1 presents the average classification accuracies obtained for LKM-Self vs. non-meditation EEG data using three session pairs based on 30 independent experiments conducted for each of the three BCI pipelines with different feature extraction methods: CSP, STFT, and a fusion of CSP and STFT. Each of these 30 experiments consists of 30 independent tests, and each row in the table shows the average accuracy and uncertainty for those 30 tests across the three BCI pipelines. For all 2700 (30 × 30 × 3) tests conducted, two session pairs were used for training the machine learning algorithm, and one session pair was used for testing the algorithm. The “Mean Accuracy (All Tests)” provides the average accuracy and uncertainty for all 900 tests conducted for each BCI pipeline. Since we observed a significant level of uncertainty for each pipeline across the 900 tests, we calculated the “Mean Accuracy (Bottom 50%)” and the “Mean Accuracy (Top 50%)” of these 900 tests. These mean accuracies and uncertainties are displayed at the bottom of Table 1.

Table 2 follows the same description as provided for Table 1, with the only difference being the use of the meditation mental task LKM-Others instead of LKM-Self. Therefore, Table 2 presents the average classification accuracies obtained for LKM-Others vs. non-meditation EEG data using three session pairs, based on 30 independent experiments conducted for each of the three BCI pipelines employing different feature extraction methods: CSP, STFT, and a fusion of CSP and STFT. Each BCI pipeline consists of 900 tests, adding up to a total of 2700 tests. The table also includes the results for “Mean Accuracy (All Tests)”, “Mean Accuracy (Bottom 50%)”, and “Mean Accuracy (Top 50%)”.

The results shown in Table 3, Table 4, Table 5 and Table 6 follow the same structure and reporting format as described for Table 1 and Table 2, with the only differences being the number of session pairs (four and five instead of three) and the meditation task (LKM-Self or LKM-Others). Each table presents the average accuracies and uncertainties across the three BCI pipelines (CSP, STFT, and CSP–STFT fusion), including “Mean Accuracy (All Tests),” “Mean Accuracy (Bottom 50%),” and “Mean Accuracy (Top 50%).”

**Figure 2:**
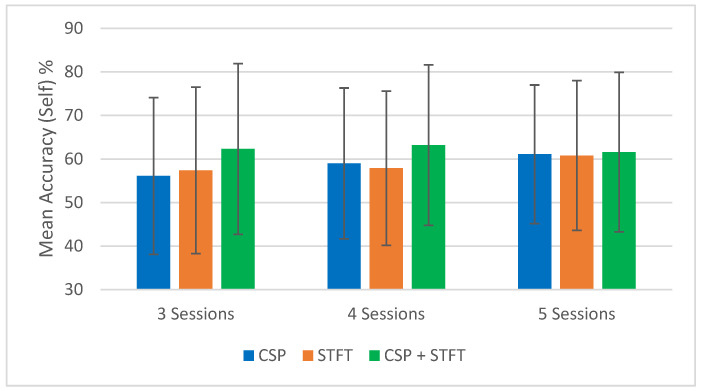
Comparison of mean classification accuracies for LKM-Self/non-meditation when using the three CSP, STFT, or CSP + STFT algorithms for the cases of 3, 4, or 5 session pairs of EEG data.

**Figure 3:**
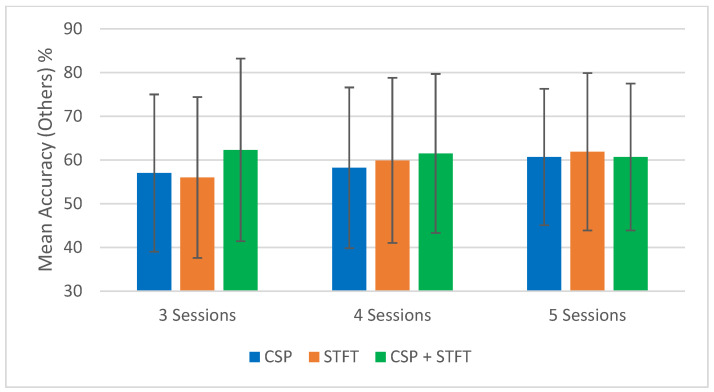
Comparison of mean classification accuracies for LKM-Others/non-meditation when using the three CSP, STFT, or CSP + STFT algorithms for the cases of 3, 4, or 5 session pairs of EEG data.

**Figure 4:**
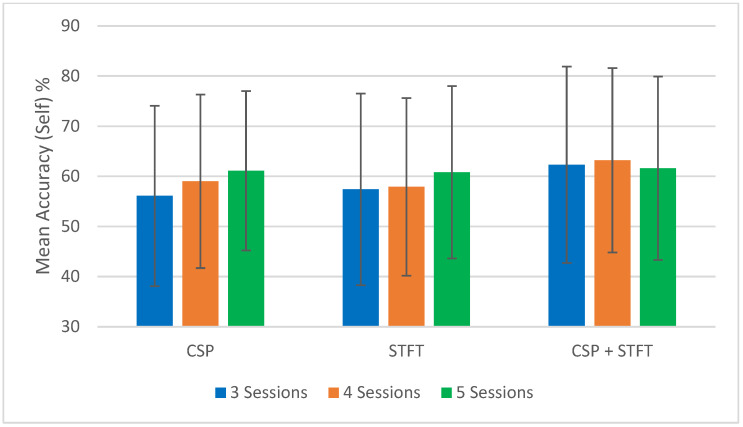
Comparison of mean classification accuracies for LKM-Self/non-meditation when using 3, 4, or 5 session pairs of EEG data for the three CSP, STFT, or CSP + STFT algorithms.

**Figure 5:**
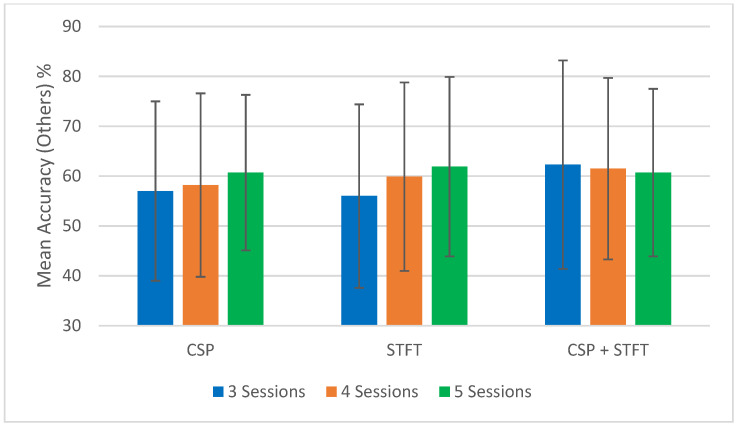
Comparison of mean classification accuracies for LKM-Others/non-meditation when using 3, 4, or 5 session pairs of EEG data for the three algorithms CSP, STFT, or CSP + STFT.

To support the tabulated results (Table 1, Table 2, Table 3, Table 4, Table 5 and Table 6), Figure 2, Figure 3, Figure 4 and Figure 5 provide a visual summary of the mean classification accuracies obtained across the three BCI pipelines (CSP, STFT, and CSP + STFT). These figures illustrate the overall performance trends for LKM-Self vs. non-meditation and LKM-Other vs. non-meditation classification under different session pair conditions (three, four, and five).

## 4. Discussion

In this study, we evaluated the performance of inter-subject (subject-independent), multi-session EEG data classification for meditation and non-meditation states and compared it with our previous work on intra-subject, multi-session EEG data classification for the same states. Six separate studies were conducted, resulting in six tables of outcomes. These included the LKM-Self vs. non-meditation study and the LKM-Others vs. non-meditation study, each performed using three, four, and five EEG session pairs. Since each of these six independent studies was tested using three different BCI pipelines, each table contains three columns corresponding to these pipelines. The main difference among the three pipelines lies in the use of different feature extraction algorithms, namely, CSP, STFT, and CSP + STFT. With three pipelines applied across the six studies, a total of 18 (6 × 3) outcomes are presented in the six result tables (Table 1, Table 2, Table 3, Table 4, Table 5 and Table 6).

In the six tables, there are 30 lines of results, and each result represents the average classification accuracy along with the corresponding error for a single random selection of five session pairs. As described in the Section 2, after randomly selecting five session pairs of meditation/non-meditation EEG data, each pipeline was tested on all possible combinations of those five pairs, with classification accuracy computed for each instance (for the three, four, and five session pairs used independently in the pipeline, the total number of possible combinations was 30, 20, and 5, respectively). These classification accuracies were then used to calculate the average classification accuracy and the associated error. This is reported as a single result in each table, and for 30 such tests, 30 results are shown for each BCI pipeline.

Our aim was to obtain a mean accuracy for each of the BCI pipelines using the results in the six tables so that the results could be compared with the mean accuracies obtained in our previous studies on intra-subject classification. Therefore, we calculated the overall average for each pipeline based on 30 independent tests. In the tables, these results are labelled as “Mean Accuracy (All Tests).” For the tables containing results of three, four, and five session pairs, these overall averages and errors for each pipeline were calculated using classification accuracy results obtained from 900, 600, and 150 independent tests, respectively. The total number of tests conducted for the entire study was 9900 ((900 + 600 + 150) × 3 × 2).

After obtaining the 18 overall mean accuracy values for the six tables, they were compared with the corresponding instances from our previous studies. We observed that the mean accuracy for each instance in this study was lower than the corresponding accuracy in the previous study for the same instance. This suggests that inter-subject classification produced lower classification accuracy than intra-subject classification. Additionally, a significant finding in this study was the high level of errors observed when calculating these mean accuracy values. These results opened a new perspective in our study, revealing that some instances yield high classification accuracies, while others yield low classification accuracies when conducting inter-subject, multi-session meditation/non-meditation classification.

Compared to our previous study, the reduced classification accuracies along with the high errors in this study demonstrate that some tests produced high classification accuracies, while others resulted in low classification accuracies. One possible explanation, which should be tested in future research, is that this variability may be related to differences in meditation experience levels among the participants, since session selection was performed randomly. Although the dataset was labelled as consisting of experienced meditators, the actual experience level, such as years of practice, was not measured. We therefore hypothesize that a range of experience levels may have existed among the so-called experienced meditation participants. If this is the case, we would expect some selection combinations to yield high classification accuracies, while others would result in lower accuracies. High accuracies could occur when the random selection included meditation EEG sessions from participants with similarly high levels of meditation experience, whereas low accuracies could result when the random selection mixed EEG data from participants with varying levels of experience.

To test this assumption for the 18 studies shown in Table 1, Table 2, Table 3, Table 4, Table 5 and Table 6, after calculating the “Mean Accuracy (All Tests)”, we also computed the “Mean Accuracy (Bottom 50%)” and “Mean Accuracy (Top 50%)”. For each BCI pipeline, the classification accuracies were divided into two equal groups, the lower 50% and the upper 50%, and the mean accuracy and corresponding error were calculated for each group. These calculations were performed across the 18 independent studies, and the resulting values are presented at the bottom of Table 1, Table 2, Table 3, Table 4, Table 5 and Table 6. The results show the mean accuracies of both the top and bottom 50% of the accuracy values obtained when testing each BCI pipeline for each of the three training sizes used in the study.

At a glance, we can observe a significant difference in classification accuracy between the mean accuracies calculated from the top and bottom halves of the results. This indicates that classification accuracy depends on the data selected for the classification. If accuracies were not influenced by the choice of sessions, the difference between the top 50% and bottom 50% would not be substantial. Since we used random selection, some combinations yielded significantly high classification accuracies, while others resulted in notably low accuracies. This was reflected in the large variance among the calculated classification accuracies for all tests within a single BCI pipeline model and further evidenced by the substantial difference between the mean accuracies of the top 50% and the bottom 50%.

**Figure 6:**
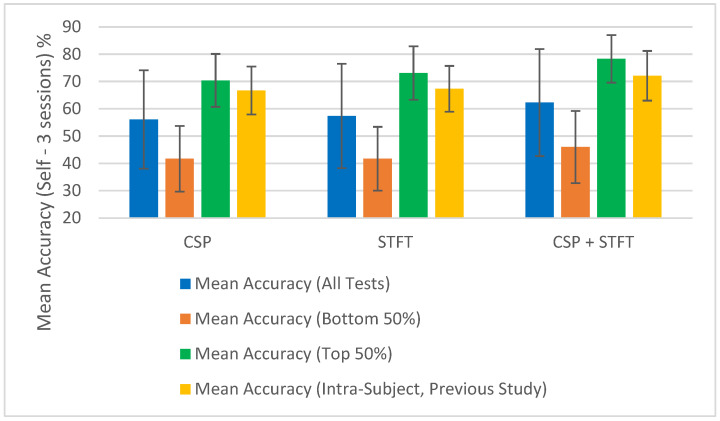
Comparison of mean inter-subject (all tests, bottom 50%, and top 50%) and intra-subject (previous study) classification accuracies for LKM-Self/non-meditation when using 3 session pairs of EEG data for the three CSP, STFT, or CSP + STFT algorithms.

**Figure 7:**
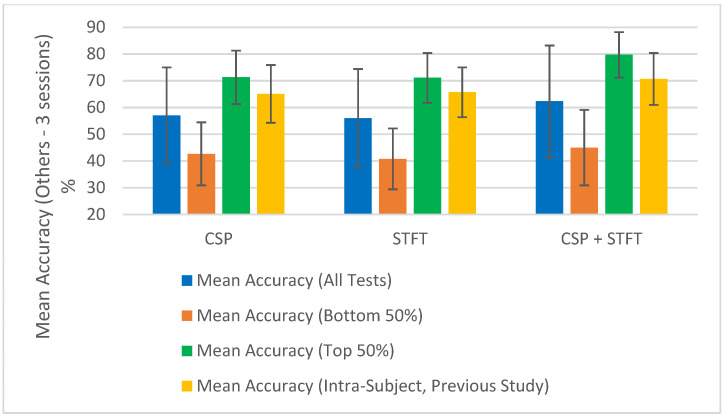
Comparison of mean inter-subject (all tests, bottom 50%, and top 50%) and intra-subject (previous study) classification accuracies for LKM-Others/non-meditation when using 3 session pairs of EEG data for the three CSP, STFT, or CSP + STFT algorithms.

**Figure 8:**
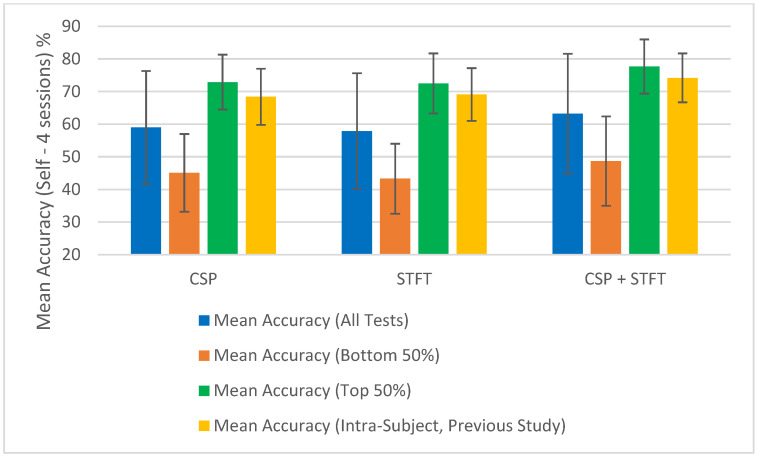
Comparison of mean inter-subject (all tests, bottom 50%, and top 50%) and intra-subject (previous study) classification accuracies for LKM-Self/non-meditation when using 4 session pairs of EEG data for the three CSP, STFT, or CSP + STFT algorithms.

**Figure 9:**
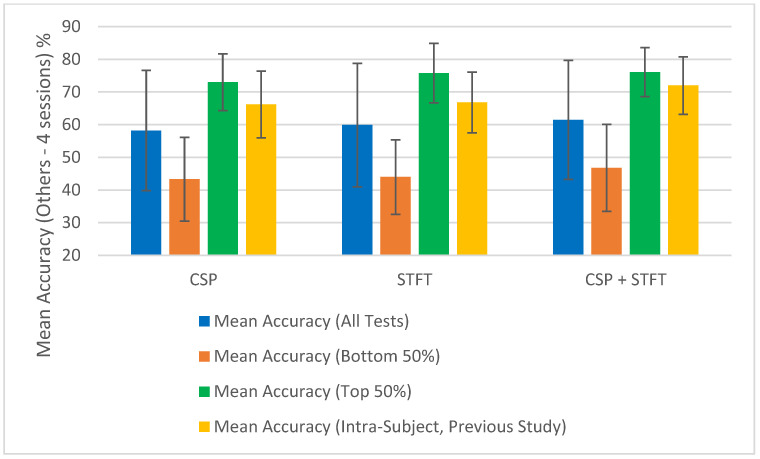
Comparison of mean inter-subject (all tests, bottom 50%, and top 50%) and intra-subject (previous study) classification accuracies for LKM-Others/non-meditation when using 4 session pairs of EEG data for the three CSP, STFT, or CSP + STFT algorithms.

**Figure 10:**
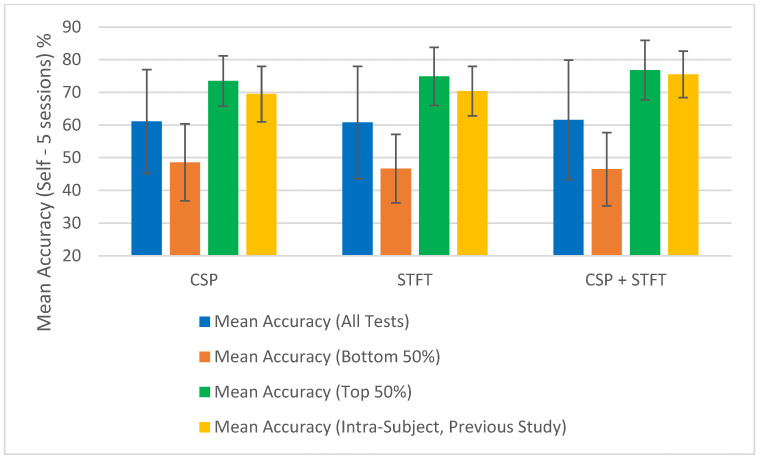
Comparison of mean inter-subject (all tests, bottom 50%, and top 50%) and intra-subject (previous study) classification accuracies for LKM-Self/non-meditation when using 5 session pairs of EEG data for the three CSP, STFT, or CSP + STFT algorithms.

**Figure 11:**
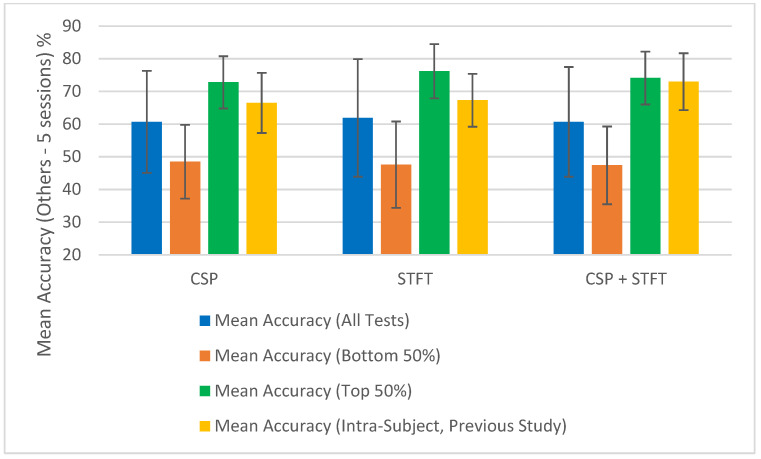
Comparison of mean inter-subject (all tests, bottom 50%, and top 50%) and intra-subject (previous study) classification accuracies for LKM-Others/non-meditation when using 5 session pairs of EEG data for the three CSP, STFT, or CSP + STFT algorithms.

The summary of the classification accuracy results (“Mean Accuracy (All Tests)”) presented in Table 1, Table 2, Table 3, Table 4, Table 5 and Table 6 is further elaborated in Figure 2, Figure 3, Figure 4 and Figure 5. Additionally, we compared the results obtained in this study (“Mean Accuracy (All Tests)”, “Mean Accuracy (Bottom 50%)”, and “Mean Accuracy (Top 50%)”) with those from our previous study, and this comparison is illustrated in Figure 6, Figure 7, Figure 8, Figure 9, Figure 10 and Figure 11. In the following sections, these figures are explained individually.

First, we focus on the performance trends of the 18 different studies, which we label as “Mean Accuracy (All Tests).” When calculating the mean accuracies, in the cases of three, four, or five session pairs, they were computed using accuracies obtained from 900, 600, and 150 tests, respectively. Figure 2, Figure 3, Figure 4 and Figure 5 present the comparison of mean classification accuracies for LKM-Self/non-meditation and LKM-Others/non-meditation using the three algorithms, CSP, STFT, and CSP + STFT, across three, four, and five session pairs of EEG data.

For LKM-Self/non-meditation (Figure 2 and Figure 4), the CSP + STFT pipeline significantly outperforms the others for three- and four-session pairs, and it performs slightly better in the five-session pair case. An increase in the number of training pairs generally improves accuracy for CSP and STFT, while for CSP + STFT, the improvement is seen mainly when comparing 3 to 4 session pairs. Overall, in 5 out of 6 cases where session pairs increased, classification accuracy improved.

For LKM-Others/non-meditation (Figure 3 and Figure 5), CSP + STFT outperforms the others in the three- and four-session pair cases, while STFT performs best in the five-session pair case. As with LKM-Self, increasing session pairs usually improved classification accuracy for CSP and STFT but not consistently for CSP + STFT. Here, 4 out of 6 cases showed improvements.

By comparing both meditation types, we see that in 9 out of 12 instances (75.0%), an increase in the number of training session pairs led to higher classification accuracy. These findings are consistent with our previous intra-subject study, where increasing training session pairs improved classification accuracy in all 12 comparisons (100%).

The performance of the three BCI pipelines (CSP, STFT, and CSP + STFT) was further tested using pairwise *t*-tests (Table 7). The results indicate that CSP + STFT performs significantly better than CSP (t (5) = −3.15, *p* = 0.025), while CSP + STFT is marginally better than STFT (t (5) = −2.43, *p* = 0.059), though not statistically significant at α = 0.05. No significant difference was found between CSP and STFT (t (5) = −0.59, *p* = 0.581).

Next, the results obtained in this study are compared with those from our previous study, and Figure 6, Figure 7, Figure 8, Figure 9, Figure 10 and Figure 11 have been prepared to provide the corresponding comparisons. The results from this study include “Mean Accuracy (All Tests)”, “Mean Accuracy (Bottom 50%)”, and “Mean Accuracy (Top 50%)”, as shown in Table 1, Table 2, Table 3, Table 4, Table 5 and Table 6. These are compared with the matching experimental results from our previous study, labelled as “Mean Accuracy (Intra-Subject, Previous Study)”. Each graph allows for a comparison of the performances of the three BCI pipelines, CSP, STFT, and CSP + STFT, for a selected meditation type and session pair count.

Figure 6 presents the classification accuracies for LKM-Self/non-meditation using 3 session pairs of EEG data with the three algorithms, CSP, STFT, or (CSP + STFT), while Figure 7 shows the corresponding results for LKM-Others/non-meditation. Similarly, Figure 8 displays the classification accuracies for LKM-Self/non-meditation using 4 session pairs, and Figure 9 presents the same for LKM-Others/non-meditation. Finally, Figure 10 and Figure 11 show the classification accuracies for LKM-Self and LKM-Others/non-meditation, respectively, when using 5 session pairs of EEG data with the three algorithms.

When we look at Figure 6, Figure 7, Figure 8, Figure 9, Figure 10 and Figure 11 for the 18 different study instances (3 BCI pipelines × 2 meditation types × 3 training session pair sizes), we observe several recurring patterns. Therefore, we will address these patterns one by one and provide a general explanation as follows.

The first observation we can elaborate on for all these 18 study instances is that the mean accuracy (all tests) in this study yielded lower classification accuracies compared to the same instances in our previous study on intra-subject classification. This is clearly understandable, as this study tested inter-subject (subject-independent) classification, meaning EEG multi-session data from different participants were used for training and testing the algorithms. In contrast, the previous study conducted a single test using multi-session data from a single person for both training and testing. This highlights that when using meditation EEG data from multiple participants, extracting meaningful information and applying it to classification algorithms becomes more complex, as such data involve greater variability than multi-session data from a single individual. Nevertheless, we were able to demonstrate that good classification performance is achievable in certain instances. Therefore, we can conclude that further research is necessary to generalize these results and achieve high mean accuracies for subject-independent classification using multiple-session meditation/non-meditation EEG data.

The second significant factor we observed in this study was that the mean accuracy (all tests) for these 18 studies exhibited significantly high error values or, in other words, variability. This suggests that the classification accuracies had a wider distribution around the mean values. What this indicates is that, while the mean accuracies in this study were lower than those in our previous study, there were individual instances that yielded relatively good accuracies, as well as those that resulted in relatively poor accuracies. These studies were conducted using a random selection of session pairs, and the high variance reveals that certain selection combinations produced good classification accuracies. Although we ensured that for each mental task, the session data were always selected from different participants, some sessions still displayed significantly higher levels of match, leading to higher classification accuracies. The underlying reasons for obtaining high accuracy for certain selection combinations and low accuracy for others remain to be explored.

One assumption we can make is that although the selected participants are labelled as expert meditators, they may still exhibit differences, falling within a range of expertise at the expert level. When the randomly selected data came from participants with similar levels of meditation skills, using session data from these participants tended to produce higher classification accuracies. Although this may or may not be the case, what remains significant is that there were individual instances that resulted in high classification accuracies. Therefore, future studies should be conducted to better understand the reasons why some session combinations fail to yield good classification accuracies and to develop solutions to address these issues. Such studies will make a meaningful contribution toward achieving subject-independent, multiple-session meditation/non-meditation classification.

To further illustrate the significance of some session combinations yielding high accuracies and others yielding low accuracies, we divided the entire accuracy result set into two equal parts and calculated the mean accuracy for each part. For each pipeline, when the number of session pairs used was three, four, and five, the number of tests conducted for each was 900, 600, and 150, respectively. In each case, this set was divided in half, with the largest half of the values placed in one group and the smallest half in another group. Then, using these selected test values, the mean and error for each group were calculated. These mean values (“Mean Accuracy (Bottom 50%)” and “Mean Accuracy (Top 50%)”) are shown in Figure 6, Figure 7, Figure 8, Figure 9, Figure 10 and Figure 11, enabling us to compare them with the full mean accuracies of this study, “Mean Accuracy (All Tests),” and the results of the past study, “Mean Accuracy (Intra-Subject, Previous Study)”.

When examining Figure 6, Figure 7, Figure 8, Figure 9, Figure 10 and Figure 11, we observe a significant difference between the mean accuracies of the bottom 50% and the top 50%, indicating that some session pair combinations yield better classification accuracies than others. Although the overall mean accuracies (all tests) in this study were lower than the corresponding values in the previous study, the mean accuracies of the top 50% were consistently higher than those of the past intra-subject classification. This further suggests that the top 50% of results in this inter-subject classification study outperformed those from the previous intra-subject study. Therefore, these positive findings highlight the importance of future research aimed at identifying the factors contributing to lower-performing combinations and improving the overall effectiveness of subject-independent, multiple-session meditation/non-meditation classification.

Additionally, we tested the results of the three-, four-, and five-session pairs independently to examine whether bimodality was present in the classification accuracies obtained in our study (Table 8). Hartigan’s dip test was used, and for the three-session case, unimodality was rejected, indicating bimodal characteristics among the 5400 test results. In contrast, the four- and five-session cases, which together consisted of 4500 tests (3600 + 900), failed to reject unimodality, indicating a lack of statistically significant bimodality. When generalizing across the entire dataset, we can conclude that although a hint of bimodality is visible, further research is needed to confirm this characteristic in multiple-session meditation/non-meditation EEG data.

Furthermore, findings that improve multiple-session classification will contribute to the future development of subject-independent EEG meditation-guiding apps. This is because progress in meditation is achieved through rigorous practice across multiple sessions, and therefore, any algorithm intended to support a person’s meditation progress should be capable of distinguishing patterns across sessions, specifically differentiating between meditation and non-meditation states. Although current research on such apps that support mental calmness and relaxation is still in its early stages, there is significant attention and high demand even for the most basic apps currently available. For these reasons, this study on subject-independent, multiple-session meditation/non-meditation classification holds great significance in current EEG research.

## 5. Limitations

This study was conducted using an EEG dataset available online, and we acknowledge some limitations connected with the dataset. Although the dataset is a large, multiple-session, high-quality EEG dataset, the number of participants is relatively low (12), and detailed characteristics such as age, gender, physical conditions, and meditation experience values are not available. Even though the dataset labels participants as “experts,” the level of meditation experience has not been independently validated, and differences within the expert group may exist. We acknowledge that these factors can have an impact on the classification accuracy in our study.

In addition, after analysis, we identified a best-performing frequency range that was applied to all participants. While this approach provided a common ground to support subject-independent classification, it did not account for individually defined frequency ranges. Since peak outcomes for each participant may slightly deviate from the generalized range, this choice may have reduced the overall classification accuracy. We therefore highlight that future studies should explore algorithms that optimize for individualized frequency ranges while still aiming for subject-independent generalization.

Finally, although multiple EEG cleaning methods were applied in EEGLAB, including bad channel removal, ICA, and visual inspection, we accept that EMG activity cannot be completely ruled out. Subtle contributions may still remain, particularly in the lower frequency bands, due to eye movements or muscle tension. Future studies should therefore consider more advanced artifact removal techniques or complementary physiological monitoring to further minimize EMG influence in EEG-based meditation classification.

## 6. Conclusions

This study explored a novel task of subject-independent (inter-subject) classification of meditation and non-meditation using multiple-session EEG data. In this research, 18 different studies, each comprising multiple tests, were conducted, totaling 9900 independent tests. The results obtained were compared with our previous study on intra-subject classification. The mean classification accuracies for inter-subject classification were lower than those for intra-subject classification, which may be due to the increased complexity of recognizing patterns when EEG data from multiple participants are used together. Nevertheless, achieving good classification accuracies for certain session combinations selected from multiple participants highlights the future potential for high-accuracy, subject-independent classification of multiple-session meditation/non-meditation EEG data. To address the limitations and challenges identified in this study, we suggest the following improvements as directions for future work.

As future work, we emphasize the importance of conducting research to achieve high classification accuracies for subject-independent, multiple-session meditation/non-meditation EEG data, which would enable the development of meditation-guiding algorithms. Future studies could also include additional evaluation metrics such as AUC and F1 scores to provide a more comprehensive assessment of classification performance. Such studies should first collect data from expert meditators with the same level of experience and skill, ideally with a sample size of around 25–30 participants, and then gather data from those with two levels of experience. Meditation expertise should be assessed using standardized experience metrics. Data from the same experience level can be used to test and improve classification accuracies, while data with two levels of experience can help identify features that distinguish between the levels. These features can then be used in studies to assess improvements in meditation practices.

When trying to identify the level of meditation experience of a person participating in EEG data collection, one method that can be used is to obtain a measurement of the duration of past meditation practice, preferably in years or months. The challenge with this approach is that some individuals may progress well in meditation within a shorter time compared to others. At the same time, an expert meditator may experience physical or mental challenges on the day of data collection, which could affect the success of the meditation session. Therefore, it is better to accompany the experience-level measurement with a psychological assessment of the meditation session, such as a questionnaire or a mindfulness and meditation scale, and to collect both types of data alongside the EEG recordings. Such data would be highly useful when studying subject-independent, multi-session EEG meditation/non-meditation classification. Furthermore, collecting data such as heart rate variability and blood oxygen level changes, in addition to EEG data during meditation/non-meditation sessions, can provide valuable features that may help achieve higher classification accuracies.

## Figures and Tables

**Table 1:** Average accuracy (%) calculated independently for each of the 30 experiments conducted for classifying LKM-Self/non-meditation EEG data using one of the three CSP, STFT, or CSP + STFT algorithms per test for the case of 3 session pairs of data.

Experiment No.	CSP	STFT	CSP + STFT
1	66.3 ± 12.0	59.5 ± 15.5	50.6 ± 25.1
2	57.7 ± 15.1	59.1 ± 10.2	77.8 ± 11.3
3	60.4 ± 9.2	47.4 ± 16.6	72.1 ± 17.7
4	61.4 ± 20.3	79.1 ± 11.0	60.1 ± 19.3
5	71.7 ± 9.0	61.0 ± 14.2	68.9 ± 8.6
6	45.3 ± 14.8	46.4 ± 15.3	77.0 ± 9.2
7	51.6 ± 16.1	72.3 ± 13.1	59.0 ± 20.1
8	63.6 ± 21.9	69.2 ± 11.9	57.5 ± 20.0
9	52.4 ± 16.0	47.5 ± 23.2	54.9 ± 15.6
10	50.2 ± 21.5	64.0 ± 14.7	57.9 ± 14.0
11	45.8 ± 15.1	55.0 ± 11.5	45.0 ± 13.8
12	56.6 ± 15.5	62.6 ± 11.7	68.6 ± 16.7
13	53.6 ± 16.4	53.7 ± 22.8	48.2 ± 22.8
14	47.8 ± 15.1	57.9 ± 16.9	57.5 ± 9.6
15	55.0 ± 12.2	48.3 ± 20.9	74.4 ± 9.8
16	63.7 ± 9.0	50.3 ± 22.8	72.0 ± 15.3
17	54.3 ± 25.4	64.0 ± 19.0	63.0 ± 26.8
18	54.9 ± 17.9	47.0 ± 21.1	80.2 ± 10.5
19	63.2 ± 17.0	65.9 ± 19.0	51.2 ± 21.0
20	54.2 ± 14.8	49.5 ± 19.6	59.8 ± 17.7
21	50.1 ± 24.1	61.5 ± 21.0	65.4 ± 11.0
22	69.9 ± 21.6	58.9 ± 14.5	63.3 ± 14.3
23	51.6 ± 10.7	54.8 ± 21.6	57.8 ± 18.4
24	59.4 ± 10.7	54.5 ± 17.1	44.5 ± 21.0
25	58.0 ± 19.4	57.8 ± 15.5	65.2 ± 13.6
26	59.4 ± 18.3	62.5 ± 17.1	78.3 ± 9.7
27	52.5 ± 10.2	55.6 ± 10.6	51.3 ± 10.8
28	47.0 ± 22.0	54.4 ± 13.3	54.5 ± 19.3
29	56.1 ± 14.1	50.9 ± 20.7	52.8 ± 18.8
30	49.0 ± 18.3	50.9 ± 24.1	79.7 ± 10.9
Mean Accuracy (All Tests)	56.1 ± 18.0	57.4 ± 19.1	62.3 ± 19.6
Mean Accuracy (Bottom 50%)	41.7 ± 12.0	41.7 ± 11.7	46.0 ± 13.2
Mean Accuracy (Top 50%)	70.4 ± 9.7	73.1 ± 9.8	78.3 ± 8.7

**Table 2:** Average accuracy (%) calculated independently for each of the 30 experiments conducted for classifying LKM-Others/non-meditation EEG data using one of the three CSP, STFT, or CSP + STFT algorithms per test for the case of 3 session pairs of data.

Experiment No.	CSP	STFT	CSP + STFT
1	67.0 ± 17.8	46.8 ± 19.7	75.1 ± 10.3
2	55.1 ± 23.9	52.7 ± 23.3	76.1 ± 10.3
3	49.9 ± 16.0	52.7 ± 14.4	52.4 ± 24.8
4	49.5 ± 24.4	50.2 ± 19.8	78.9 ± 17.9
5	67.0 ± 14.2	60.0 ± 12.6	78.1 ± 14.8
6	45.7 ± 15.7	49.2 ± 20.4	78.1 ± 13.5
7	63.7 ± 24.0	51.2 ± 14.3	45.2 ± 24.1
8	54.7 ± 11.3	52.6 ± 19.1	63.1 ± 27.2
9	67.7 ± 20.5	54.7 ± 19.7	66.9 ± 18.9
10	50.7 ± 17.0	57.1 ± 18.2	58.2 ± 12.2
11	58.3 ± 10.3	54.3 ± 17.6	50.4 ± 15.8
12	68.0 ± 8.8	57.1 ± 19.1	51.0 ± 22.5
13	55.7 ± 20.7	56.0 ± 16.9	76.4 ± 8.5
14	52.5 ± 16.8	58.3 ± 19.8	56.6 ± 23.1
15	51.0 ± 15.9	67.0 ± 13.3	53.7 ± 18.5
16	56.8 ± 6.4	50.9 ± 17.4	55.9 ± 15.8
17	46.6 ± 19.8	62.2 ± 16.0	65.9 ± 18.6
18	53.8 ± 9.2	47.7 ± 20.7	60.4 ± 18.5
19	52.5 ± 14.6	56.9 ± 17.0	58.5 ± 15.2
20	58.1 ± 15.7	59.7 ± 14.6	72.9 ± 18.9
21	50.2 ± 19.5	43.4 ± 21.5	64.5 ± 14.0
22	65.9 ± 16.3	60.8 ± 15.3	64.1 ± 20.1
23	56.9 ± 16.9	70.8 ± 8.4	54.9 ± 16.3
24	56.1 ± 15.7	53.7 ± 21.9	53.4 ± 27.7
25	63.7 ± 13.5	61.6 ± 14.5	53.3 ± 15.6
26	65.1 ± 16.0	66.7 ± 10.6	66.6 ± 19.6
27	53.8 ± 17.2	56.9 ± 17.6	62.7 ± 18.8
28	62.0 ± 24.1	47.5 ± 14.2	61.0 ± 17.5
29	59.8 ± 9.3	60.6 ± 18.4	51.8 ± 24.5
30	51.9 ± 11.8	59.0 ± 10.7	63.2 ± 17.0
Mean Accuracy (All Tests)	57.0 ± 18.0	56.0 ± 18.4	62.3 ± 20.9
Mean Accuracy (Bottom 50%)	42.7 ± 11.8	40.8 ± 11.4	45.0 ± 14.1
Mean Accuracy (Top 50%)	71.3 ± 10.0	71.1 ± 9.3	79.7 ± 8.5

**Table 3:** Average accuracy (%) calculated independently for each of the 30 experiments conducted for classifying LKM-Self/non-meditation EEG data using one of the three CSP, STFT, or CSP + STFT algorithms per test for the case of 4 session pairs of data.

Experiment No.	CSP	STFT	CSP + STFT
1	67.4 ± 11.4	44.7 ± 10.4	62.0 ± 12.5
2	57.9 ± 16.8	52.3 ± 17.7	72.1 ± 19.4
3	67.2 ± 19.4	53.4 ± 19.1	60.1 ± 28.0
4	69.0 ± 22.2	66.5 ± 19.6	71.4 ± 10.4
5	62.5 ± 10.8	65.6 ± 18.5	54.3 ± 21.9
6	55.9 ± 18.1	57.5 ± 16.3	58.4 ± 12.1
7	65.8 ± 11.9	58.9 ± 19.0	54.7 ± 8.3
8	61.6 ± 14.2	69.0 ± 9.2	46.4 ± 18.7
9	52.0 ± 15.2	49.7 ± 18.0	58.6 ± 25.5
10	60.0 ± 16.9	53.2 ± 15.0	57.1 ± 16.4
11	62.9 ± 19.7	52.7 ± 15.6	68.8 ± 13.2
12	57.9 ± 15.6	44.8 ± 11.0	63.2 ± 12.9
13	65.1 ± 11.7	61.5 ± 19.9	63.2 ± 10.6
14	54.0 ± 17.4	62.8 ± 16.9	71.2 ± 20.3
15	52.0 ± 15.2	52.4 ± 18.5	55.4 ± 25.0
16	58.2 ± 20.3	55.2 ± 16.9	47.4 ± 11.3
17	49.8 ± 12.0	56.5 ± 15.4	49.6 ± 17.1
18	53.2 ± 17.9	56.7 ± 12.2	69.6 ± 15.1
19	52.3 ± 23.1	54.8 ± 24.3	55.4 ± 16.6
20	52.5 ± 14.0	66.0 ± 15.0	68.7 ± 12.9
21	53.2 ± 17.0	50.2 ± 22.9	66.4 ± 14.3
22	57.4 ± 11.9	55.1 ± 16.1	62.6 ± 22.8
23	64.8 ± 14.5	67.1 ± 12.1	79.1 ± 8.6
24	56.2 ± 15.9	46.0 ± 13.4	58.1 ± 16.2
25	68.0 ± 8.6	62.6 ± 14.2	70.8 ± 12.7
26	56.0 ± 22.7	62.3 ± 15.3	72.8 ± 7.5
27	66.0 ± 8.4	63.0 ± 14.9	65.1 ± 12.6
28	59.2 ± 9.2	54.2 ± 11.5	73.8 ± 8.2
29	67.0 ± 17.0	75.0 ± 9.7	64.0 ± 19.5
30	44.5 ± 19.3	67.0 ± 10.2	76.8 ± 13.0
Mean Accuracy (All Tests)	59.0 ± 17.3	57.9 ± 17.7	63.2 ± 18.4
Mean Accuracy (Bottom 50%)	45.1 ± 11.9	43.3 ± 10.7	48.7 ± 13.7
Mean Accuracy (Top 50%)	72.9 ± 8.4	72.5 ± 9.2	77.7 ± 8.3

**Table 4:** Average accuracy (%) calculated independently for each of the 30 experiments conducted for classifying LKM-Others/non-meditation EEG data using one of the three CSP, STFT, or CSP + STFT algorithms per test for the case of 4 session pairs of data.

Experiment No.	CSP	STFT	CSP + STFT
1	76.4 ± 6.8	42.6 ± 11.1	75.8 ± 7.4
2	43.3 ± 15.1	72.5 ± 15.3	68.9 ± 25.6
3	61.4 ± 25.4	50.3 ± 15.7	63.6 ± 18.9
4	45.2 ± 22.5	43.5 ± 17.2	63.4 ± 20.3
5	75.6 ± 20.7	48.6 ± 16.7	70.8 ± 10.3
6	45.6 ± 17.2	62.9 ± 24.0	56.2 ± 20.8
7	63.9 ± 14.5	81.0 ± 10.2	55.2 ± 17.3
8	68.0 ± 8.6	78.9 ± 15.5	44.8 ± 15.4
9	47.8 ± 14.0	63.9 ± 19.2	55.3 ± 17.8
10	69.4 ± 7.2	40.5 ± 14.7	53.6 ± 16.3
11	57.2 ± 19.6	54.7 ± 14.1	65.2 ± 19.3
12	67.4 ± 18.4	75.5 ± 14.5	70.1 ± 12.3
13	55.8 ± 16.7	54.3 ± 10.8	60.4 ± 18.8
14	49.4 ± 14.5	53.5 ± 13.3	64.2 ± 15.9
15	56.4 ± 18.0	59.7 ± 17.7	65.5 ± 15.7
16	70.0 ± 9.7	78.0 ± 10.4	51.6 ± 14.0
17	41.6 ± 14.4	54.4 ± 14.3	69.2 ± 8.0
18	66.7 ± 7.7	62.3 ± 12.0	63.3 ± 16.6
19	43.6 ± 12.6	68.1 ± 12.4	70.2 ± 17.9
20	48.8 ± 15.1	64.2 ± 13.1	65.5 ± 11.7
21	64.6 ± 16.4	68.8 ± 20.2	57.3 ± 16.0
22	51.2 ± 14.3	62.3 ± 15.8	57.6 ± 14.2
23	70.3 ± 12.5	54.5 ± 19.5	69.3 ± 13.1
24	56.8 ± 13.6	46.9 ± 14.9	43.1 ± 16.3
25	60.1 ± 19.1	72.7 ± 10.7	52.2 ± 19.9
26	68.4 ± 10.8	66.9 ± 13.3	72.4 ± 10.4
27	57.5 ± 10.5	61.9 ± 13.6	42.9 ± 15.9
28	42.7 ± 16.6	45.2 ± 16.7	60.4 ± 11.3
29	63.8 ± 15.9	59.3 ± 16.6	62.9 ± 16.9
30	56.1 ± 12.7	49.8 ± 14.7	72.7 ± 9.5
Mean Accuracy (All Tests)	58.2 ± 18.4	59.9 ± 18.9	61.5 ± 18.2
Mean Accuracy (Bottom 50%)	43.3 ± 12.8	44.0 ± 11.4	46.8 ± 13.3
Mean Accuracy (Top 50%)	73.0 ± 8.7	75.8 ± 9.1	76.1 ± 7.5

**Table 5:** Average accuracy (%) calculated independently for each of the 30 experiments conducted for classifying LKM-Self/non-meditation EEG data using one of the three CSP, STFT, or CSP + STFT algorithms per test for the case of 5 session pairs of data.

Experiment No.	CSP	STFT	CSP + STFT
1	66.1 ± 12.4	36.5 ± 12.6	36.3 ± 12.2
2	52.9 ± 12.1	66.9 ± 15.0	52.5 ± 18.3
3	63.6 ± 8.3	66.5 ± 12.6	72.0 ± 8.6
4	66.5 ± 17.0	79.8 ± 14.6	72.5 ± 9.8
5	62.9 ± 9.1	57.2 ± 14.2	73.3 ± 13.5
6	60.9 ± 15.8	63.3 ± 14.0	51.2 ± 14.0
7	57.4 ± 16.2	55.0 ± 9.5	57.6 ± 19.1
8	57.0 ± 12.5	74.5 ± 16.1	74.9 ± 12.9
9	61.2 ± 9.9	61.1 ± 18.7	43.4 ± 22.4
10	63.4 ± 18.3	54.4 ± 16.1	57.6 ± 16.8
11	72.0 ± 9.4	65.8 ± 8.6	42.3 ± 10.0
12	61.0 ± 22.0	66.8 ± 16.6	55.6 ± 15.8
13	60.7 ± 16.0	59.3 ± 12.5	71.9 ± 11.2
14	69.4 ± 13.3	53.9 ± 15.5	74.7 ± 9.5
15	58.4 ± 10.4	52.6 ± 10.1	70.3 ± 17.4
16	68.1 ± 20.0	66.2 ± 18.6	56.7 ± 18.3
17	57.7 ± 10.8	71.3 ± 19.4	75.0 ± 16.5
18	38.7 ± 14.2	56.1 ± 8.8	87.5 ± 8.5
19	56.0 ± 15.7	69.8 ± 6.8	71.3 ± 20.4
20	48.1 ± 15.7	66.2 ± 13.1	49.6 ± 12.1
21	56.6 ± 3.6	45.8 ± 9.0	63.2 ± 7.6
22	58.8 ± 4.3	48.0 ± 9.3	61.5 ± 20.0
23	60.4 ± 26.3	62.4 ± 13.9	43.9 ± 4.0
24	63.8 ± 11.8	50.8 ± 19.1	74.0 ± 15.2
25	71.9 ± 12.9	63.4 ± 11.1	62.7 ± 11.0
26	64.5 ± 12.7	72.1 ± 10.0	59.3 ± 6.6
27	50.0 ± 11.3	50.8 ± 21.2	67.9 ± 10.2
28	73.6 ± 14.7	71.9 ± 9.3	50.7 ± 11.8
29	63.6 ± 13.8	58.7 ± 15.2	62.8 ± 11.8
30	66.8 ± 10.2	57.1 ± 22.8	57.4 ± 7.2
Mean Accuracy (All Tests)	61.1 ± 15.9	60.8 ± 17.2	61.6 ± 18.3
Mean Accuracy (Bottom 50%)	48.6 ± 11.8	46.7 ± 10.5	46.5 ± 11.2
Mean Accuracy (Top 50%)	73.5 ± 7.7	74.9 ± 8.9	76.8 ± 9.1

**Table 6:** Average accuracy (%) calculated independently for each of the 30 experiments conducted for classifying LKM-Others/non-meditation EEG data using one of the three CSP, STFT, or CSP + STFT algorithms per test for the case of 5 session pairs of data.

Experiment No.	CSP	STFT	CSP + STFT
1	65.7 ± 14.0	71.7 ± 7.0	51.2 ± 17.8
2	60.6 ± 21.3	64.4 ± 12.0	61.7 ± 12.4
3	71.2 ± 12.0	54.9 ± 13.4	54.2 ± 17.6
4	71.8 ± 18.7	70.7 ± 15.2	54.5 ± 13.9
5	58.6 ± 12.6	49.8 ± 16.7	51.5 ± 17.3
6	55.5 ± 21.1	56.4 ± 6.1	60.9 ± 11.9
7	66.9 ± 6.7	49.7 ± 16.6	55.9 ± 12.2
8	51.1 ± 8.4	66.2 ± 17.7	64.6 ± 15.3
9	56.8 ± 8.4	67.6 ± 13.0	39.5 ± 7.6
10	57.5 ± 19.3	73.5 ± 11.8	77.5 ± 10.8
11	43.8 ± 8.9	44.3 ± 16.0	65.8 ± 10.3
12	57.4 ± 13.2	69.2 ± 15.9	69.7 ± 9.1
13	60.9 ± 13.2	56.4 ± 22.1	73.0 ± 9.1
14	70.1 ± 11.7	51.7 ± 14.4	74.0 ± 10.1
15	53.0 ± 8.8	56.2 ± 19.0	52.4 ± 13.6
16	48.7 ± 14.9	66.0 ± 16.2	61.4 ± 16.7
17	57.4 ± 18.4	70.5 ± 22.6	58.0 ± 11.2
18	41.9 ± 16.8	67.3 ± 23.9	73.9 ± 9.2
19	73.2 ± 10.1	59.7 ± 9.9	63.0 ± 20.0
20	62.6 ± 6.6	77.9 ± 11.5	29.1 ± 10.0
21	52.7 ± 4.8	69.6 ± 7.0	51.6 ± 13.7
22	63.7 ± 13.0	56.2 ± 7.0	57.9 ± 9.4
23	70.8 ± 6.0	54.3 ± 21.2	71.4 ± 19.2
24	63.1 ± 10.0	53.8 ± 26.0	77.1 ± 7.3
25	66.4 ± 16.0	52.2 ± 24.2	50.3 ± 12.6
26	71.9 ± 13.9	65.8 ± 12.2	71.5 ± 8.8
27	49.4 ± 13.6	54.2 ± 9.8	57.4 ± 17.5
28	64.1 ± 6.5	74.8 ± 9.8	63.5 ± 8.6
29	66.0 ± 9.3	67.2 ± 9.1	67.2 ± 6.8
30	66.7 ± 11.3	65.0 ± 17.5	62.2 ± 7.5
Mean Accuracy (All Tests)	60.7 ± 15.6	61.9 ± 18.0	60.7 ± 16.8
Mean Accuracy (Bottom 50%)	48.5 ± 11.3	47.6 ± 13.2	47.4 ± 11.9
Mean Accuracy (Top 50%)	72.8 ± 8.0	76.2 ± 8.3	74.1 ± 8.1

**Table 7:** Paired *t*-test results comparing classification accuracies across methods (d.f. = 5 for all comparisons).

Comparison	t-Stat	*p*-Value
CSP vs. CSP + STFT	−3.15	0.025
STFT vs. CSP + STFT	−2.43	0.059
CSP vs. STFT	−0.59	0.581

Note: *p* = 0.059 indicates a trend but not statistical significance at α = 0.05.

**Table 8:** Hartigan’s dip test results for classification accuracy distributions across different session-pair conditions.

Session Pair Type	No. of Tests	Dip Statistic	*p*-Value
3-session pairs	5400	0.0077	0.0347
4-session pairs	3600	0.0085	0.0831
5-session pairs	900	0.0152	0.1793

## Data Availability

The dataset DOI is “10.18112/openneuro.ds003816.v1.0.1” and is available at https://openneuro.org/datasets/ds003816/versions/1.0.1 (accessed on 13 January 2022).
